# Influence of Titanium on Microstructure, Phase Formation and Wear Behaviour of AlCoCrFeNiTi_x_ High-Entropy Alloy

**DOI:** 10.3390/e20070505

**Published:** 2018-07-02

**Authors:** Martin Löbel, Thomas Lindner, Thomas Mehner, Thomas Lampke

**Affiliations:** Materials and Surface Engineering Group, Institute of Materials Science and Engineering, Chemnitz University of Technology, D-09125 Chemnitz, Germany

**Keywords:** HEA, high-entropy alloy, CCA, compositionally complex alloy, phase composition, microstructure, wear behaviour

## Abstract

The novel alloying concept of high-entropy alloys (HEAs) has been the focus of many recent investigations revealing an interesting combination of properties. Alloying with aluminium and titanium showed strong influence on microstructure and phase composition. However, detailed investigations on the influence of titanium are lacking. In this study, the influence of titanium in the alloy system AlCoCrFeNiTi_x_ was studied in a wide range (molar ratios x = 0.0; 0.2; 0.5; 0.8; 1.0; 1.5). Detailed studies investigating the microstructure, chemical composition, phase composition, solidification behaviour, and wear behaviour were carried out. Alloying with titanium showed strong influence on the resulting microstructure and lead to an increase of microstructural heterogeneity. Phase analyses revealed the formation of one body-centred cubic (bcc) phase for the alloy without titanium, whereas alloying with titanium caused the formation of two different bcc phases as main phases. Additional phases were detected for alloys with increased titanium content. For x ≥ 0.5, a minor phase with face-centred cubic (fcc) structure was formed. Further addition of titanium led to the formation of complex phases. Investigation of wear behaviour revealed a superior wear resistance of the alloy AlCoCrFeNiTi_0.5_ as compared to a bearing steel sample.

## 1. Introduction

High-entropy alloys (HEAs) are an emerging class of new materials. Their alloying concept differs from conventional alloys, which are composed of one main element, and an improvement of properties is achieved by adding minor amounts of other elements. In contrast, HEAs are multicomponent alloys comprising at least five elements with approximately equimolar composition [1]. Despite their complex composition, only simple solid solutions with fcc or bcc structure were formed for several alloy systems. The formation of brittle and intermetallic phases can be successfully suppressed. One of the first alloys with a single fcc phase is the equimolar alloy CoCrFeMnNi investigated by Cantor et al. [2]. Due to their unique structure, HEAs exhibit an interesting combination of properties e.g., high hardness and strength in combination with adequate ductility. Furthermore, a high wear and corrosion resistance can be obtained [3,4,5,6]. Two main groups can be distinguished: HEAs forming (i) cubic or (ii) hexagonal phases [4].

Detailed structural investigations of HEAs revealed that only a few alloys form a single phase microstructure comprising fcc or bcc phases. Most alloys are composed of more than one phase, partly including complex or intermetallic phases. For these alloys, the term compositionally complex alloys (CCA) was introduced [7].

One of the most intensely investigated HEA systems, primarily forming cubic phases is AlCoCrCuFeNi [8,9]. Due to the positive enthalpy of mixing ∆H_mix_ among Cu and the elements Fe and Cr, segregation was observed, deteriorating mechanical and corrosion properties. Therefore, subsequent investigations focused on the copper-free derivative AlCoCrFeNi [3,10,11,12,13]. Early studies concentrated on the equimolar composition. However, investigations showed that optimum properties are usually achieved when choosing a differing chemical composition [14].

Investigating the influence of different alloying elements showed a strong influence of aluminium on the microstructure, phase composition, and properties [15,16]. For a low aluminium content, fcc phases are stabilised, whereas high contents of aluminium act as a strong bcc phase stabiliser [17,18]. In addition, the formation of complex phases can be suppressed for a high aluminium content [19].

Furthermore, the influence of additional alloying elements has been investigated. One element which shows distinct effects on microstructure, phase composition, and mechanical properties is titanium. Due to its large atomic radius, titanium leads to solid solution strengthening, increasing its hardness and strength. However, for a high titanium content, the formation of intermetallic phases and compounds was determined, leading to embrittlement. The alloy system AlCoCrFeNiTi shows high wear resistance also in comparison to conventional steels [20,21,22,23,24].

Detailed investigations on the influence of the alloying element titanium are required for the development of lightweight HEAs. The aim of the present study is the determination of the influence of this alloying element in the alloy system AlCoCrFeNiTi_x_ regarding its influence on microstructure, phase composition and wear behaviour.

## 2. Materials and Methods

Bulk samples of the alloy system AlCoCrFeNiTi_x_ with the molar ratios of x = 0.0; 0.2; 0.5; 0.8; 1.0; 1.5 were produced by arc-melting. Elemental granules with a purity of ≥99.9% were used as raw materials. The elemental granules were weighed and mixed according to the intended molar ratios. Each sample had a total weight of 10 g. Arc-melting of the samples was conducted in a water-cooled copper crucible.

After evacuating and reaching a pressure of 2 × 10^−4^ mbar, the furnace chamber was filled with argon to a pressure of 1.1 bar. A tungsten electrode was used to ignite an arc. All samples were remoulded three times and turned after each step to achieve chemical homogeneity. The resulting samples had a diameter of approximately 20 mm. For the arc furnace, a low cooling rate of <50 K/s was determined in preliminary studies.

Metallographic cross-sections were prepared according to standard metallographic procedures. Investigations of the microstructure were carried out by scanning electron microscopy (SEM) in a LEO 1455VP (Zeiss, Jena, Germany) with an acceleration voltage of 25 kV. For the visualisation of material contrast, a backscattered-electron detector (BSD) was used. The analyses of the overall chemical composition was carried out by energy dispersive X-ray spectroscopy (EDS) with a GENESIS spectrometer (EDAX, Mahwah, NJ, USA) at a magnification of 500× within an analysis area of approximately 43,500 µm^2^. Three measurements were carried out for every sample. Microhardness measurements (Vickers hardness HV0.5) were conducted with a Wilson Tukon 1102 device (Buehler, Uzwil, Switzerland) in metallographic cross-sections. The average microhardness and standard deviation was calculated from ten single indents. Phase analyses was conducted by X-ray diffraction (XRD) with a D8 Discover diffractometer (Bruker AXS, Billerica, MA, USA) using Co Kα radiation (tube parameters: 40 kV; 40 mA). The diffractograms were measured in a diffraction angle range (2θ) of 20° to 130° with a step size of 0.01° and 3.4 s/step, which corresponds to 653 s/step due to the utilisation of a 1D Lynxeye XE detector. For phase identification, the powder diffraction file (PDF) database 2014 (International Centre for Diffraction Data) was used. The solidification behaviour was investigated by differential scanning calorimetry (DSC) with a STA 409 C device (Netzsch, Selb, Germany) under argon atmosphere in the temperature range from 1800 K to room temperature with a cooling rate of 20 K/min.

To investigate the tribological behaviour under adhesive, oscillating, and abrasive wear conditions, ball-on-disk, oscillating wear and scratch tests have been carried out. For the ball-on-disk tests a Tetra Basalt Tester (Tetra, Ilmenau, Germany) were used. The oscillating wear tests were carried out with a Wazau SVT 40 device (Wazau, Berlin, Germany) and a CSM Revetest-RST device (CSM Instruments SA, Peseux, Switzerland) has been used for the scratch tests. The applied parameters are summarised in Table 1.

The measurements of the resulting wear depths after the ball-on-disk test were conducted by a contact stylus instrument with a Hommel Etamic T8000 device (Jenoptik, Villingen-Schwenningen, Germany). Resulting wear marks of the oscillating wear and scratch tests were analysed by laser scanning microscopy (LSM) with a Keyence VK-X200 device (Keyence, Osaka, Japan) to determine the resulting wear depth. The reference material bearing steel EN 1.3505 (100Cr6) was investigated under identical conditions. Wear marks of the scratch test were investigated with the digital microscope Keyence VHX-500 (Keyence, Osaka, Japan).

## 3. Results and Discussion

### 3.1. Chemical Composition

The mean chemical composition of all samples was measured. Results are summarised in Table 2.

The chemical composition of the arc-melted samples was in good agreement with the nominal values. Only for the aluminium content did a distinct deviation of >1 at.% occur. The titanium content was in good agreement with the nominal values for all samples.

### 3.2. Microstructure

**Figure 1:**
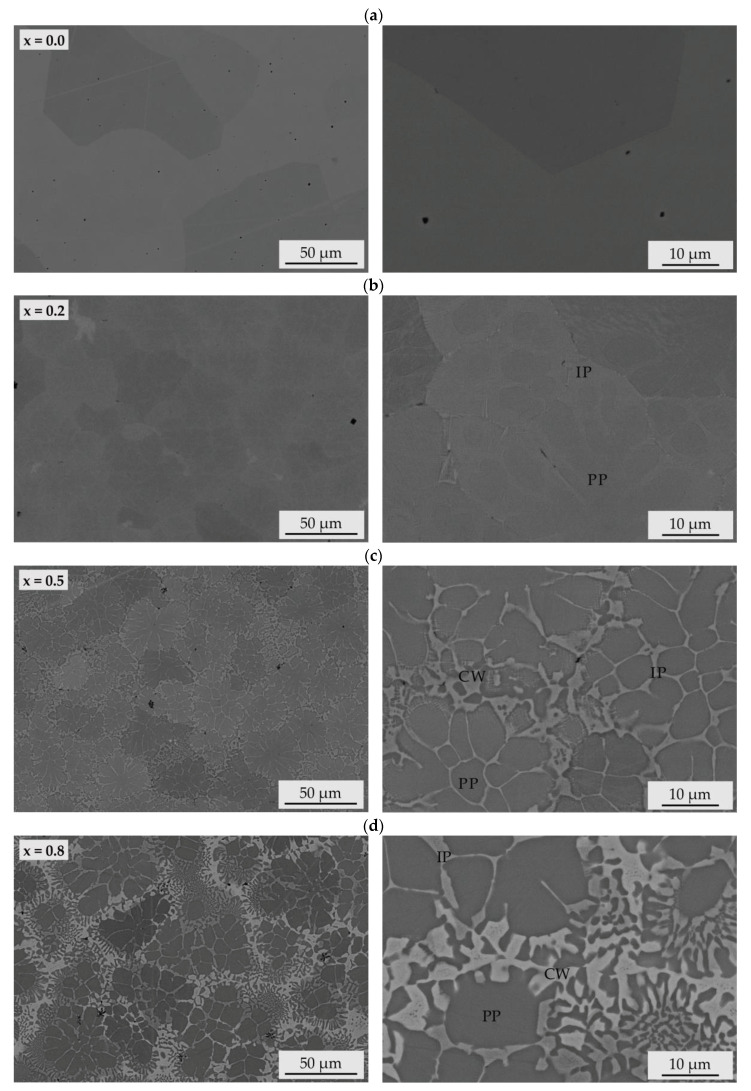

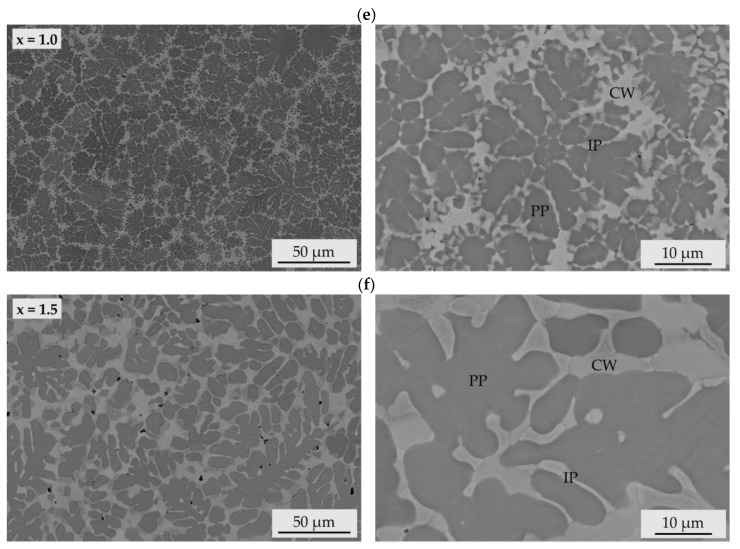
SEM micrographs (BSD detector) of arc-melted AlCoCrFeNiTi_x_ samples: (**a**) x = 0.0; (**b**) x = 0.2; (**c**) x = 0.5; (**d**) x = 0.8; (**e**) x = 1.0; (**f**) x = 1.5 with phase declaration (PP: primary phase; CW: cell wall; IP: interdendritic phase). The formation of additional phases and an increase of heterogeneity in the microstructure can be observed for an increased titanium content.

SEM images of the microstructure using a BSD to visualise material contrast with different magnifications are shown in Figure 1.

For the alloy without titanium (x = 0.0), a homogeneous microstructure occurred. The grains solidified with no preferred direction. Low differences of the chemical composition were confirmed by a minor BSD contrast. Alloying with titanium causes the formation of a more heterogeneous microstructure. Within the grains, material contrast was observed for the alloy with x = 0.2, indicating differences in the chemical composition. Between both of these areas, no distinct boundaries occurred, indicating a directional solidification with minor change in orientation. During the solidification, the precipitation of the primary phase caused a depletion of alloy elements in the residual liquid phase, causing the precipitation of a secondary phase with a different chemical composition. With further increased titanium content (x = 0.5), a dendritic structure appeared. A bright-appearing phase (interdendritic phase) was observed at the grain boundaries of the primary phase, indicating that this area is rich in elements with a high atomic number. A third phase (cell wall) solidifies as a remainder between the dendritic structures. For the alloy with higher titanium content (x = 0.8), the content of this third phase increased. Also, for the samples with further increased titanium content (x = 1.0 and x = 1.5), a dendritic structure comprising three distinguishable phases appeared. The cross-sections of all arc-melted samples exhibited cavities and shrinkage porosity caused by a different contraction of the present phases, and small amounts of breakouts due to the metallographic preparation or sample production (black areas). The presence of these defects was most distinct for the alloy with the highest titanium content (x = 1.5).

### 3.3. Solidification Behaviour and Phase Analyses

**Figure 2:**
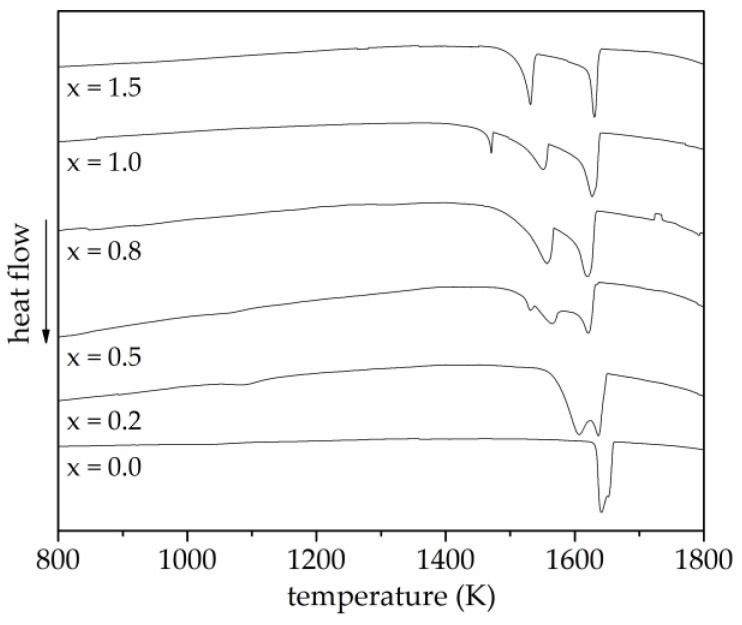
DSC cooling curves of arc-melted AlCoCrFeNiTi_x_ samples. A single exothermic reaction can be observed for the alloy without titanium, whereas several exothermic reactions occur for all alloys containing titanium, revealing the formation of additional phases.

The solidification behaviour was determined by DSC measurements. The resulting cooling curves in a temperature range of 1800 K to 800 K are shown in Figure 2.

The DSC cooling curve of the alloy without titanium (x = 0.0) exhibited one peak at a temperature of 1640 K. All investigated alloys showed a major peak at a temperature above 1600 K, corresponding to the major primarily formed phase. For the alloy with (x = 0.2), two distinct exothermic reactions occurred, showing that two major phases were formed. The similar temperature range indicated a continuous solidification. For the alloys with increased titanium content (x = 0.5 and 0.8), the DSC cooling curves also showed two major exothermic reactions. However, the increasing temperature shift of the peaks indicated a changed solidification behaviour and chemical composition of the second major phases, while the primary phase precipitated at the same temperature. For the equimolar alloy (x = 1.0), a further peak appeared at a temperature of 1470 K, showing that an additional phase was formed. The DSC cooling curve of the alloy with the highest titanium content (x = 1.5) only displayed two exothermic reactions, which can be ascribed to the two major phases also detected for the alloys with lower titanium content. No further peak of another phase visible in the SEM images (Figure 1f) appeared, indicating a small phase content or a similar solidification temperature.

**Figure 3:**
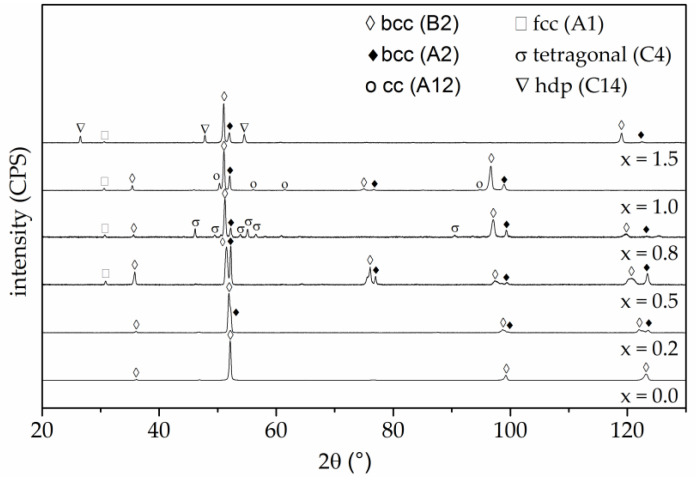
XRD diffractograms of arc-melted AlCoCrFeNiTi_x_ samples. A single phase is formed for the alloy without titanium, whereas diffraction peaks of additional phases occur for an increased titanium content. Complex phases are formed for x ≥ 0.8.

For the assignment of phases, the resulting diffractograms of the XRD phase analyses are shown in Figure 3.

The diffraction diagram of the alloy without titanium (x = 0.0) exhibited high intensity diffraction peaks which could be ascribed to a chemically ordered bcc phase with B2 structure. Diffraction peaks of this phase appeared for all investigated alloys, which was in accordance with DSC results, revealing that this phase is the primary phase (PP). For all titanium containing alloys, the diffractograms showed an additional phase with a bcc structure: a chemically disordered bcc phase with A2 structure. This phase corresponded to the second exothermic reaction in the DSC measurements, and hence to the interdendritic phase (IP) in the alloys x ≥ 0.2. The stabilisation of bcc phases due to a high aluminium content has been reported elsewhere in detail [19]. For the alloy with the lowest titanium content (x = 0.2), no additional phases were be detected. However, with increased titanium content further diffraction peaks occurred. For all alloys with a titanium content of x ≥ 0.5, an additional peak at a diffraction angle of 31.0° appeared. This diffraction angle could be ascribed to an fcc phase. Further diffraction peaks of this phase overlapped with the bcc (B2) phase. Previous investigations of the alloy system Al_x_CoCrFeNiTi revealed the solidification of the fcc phase as a remainder in the cell walls [19]. The diffraction diagram of the alloy with a titanium content of x = 0.8 exhibited several additional diffraction peaks, which can be assigned to a tetragonal σ phase. This phase has also been detected by Moravcik et al. for the alloy AlCoCrFeNiTi_0.5_ produced by spark plasma sintering (SPS). Microstructural investigations revealed the formation of this phase embedded in a mixture of other phases around a primary phase. However, subsequent heat treatment resulted in the dissolution of the σ phase, showing that the formation of this phase sensitively depends on manufacturing conditions [25]. For the equimolar alloy (x = 1.0), no diffraction peaks of the σ phase appeared. Additional peaks occurred, which can be ascribed to a centred cluster (cc) with A12 structure type. DSC results also revealed the formation of an additional phase, which solidifies as a remainder after the two major bcc phases and forms cell walls in the microstructure. This phase has been detected in the same alloy system in preliminary studies by Lindner et al. and for a high aluminium content (AlCoCrFeNiTi_1.5_), the formation of this phase could be suppressed [19]. The diffractogram of the alloy with the highest titanium content (x = 1.5) exhibited diffraction peaks which can be ascribed to the bcc (A2), bcc (B2) and an fcc phase. However, the diffraction peaks at 2θ = 35.4°; 96.7° and 98.9° of these phases did not appear. This might be caused by the relatively coarse microstructure and texture of the samples due to the comparatively slow cooling conditions. The diffraction diagram did not exhibit peaks of the σ or cc (A12) phase. Additional diffraction peaks can be ascribed to a hexagonal Laves phase (C14/MgZn_2_ type). A similar phase with slightly changed lattice parameters has been detected by Zhou et al. [20]. The present phases and corresponding major crystallographic information are summarised in Table 3.

In addition to the formation of further phases, a shift of lattice parameters was observed with increasing titanium content. The lattice parameters of the bcc (B2) phase and the fcc (A1) phase were slightly increased. This behaviour indicated that more titanium with a large atomic radius was resolved in these phases.

### 3.4. Hardness and Wear Behaviour

**Figure 4:**
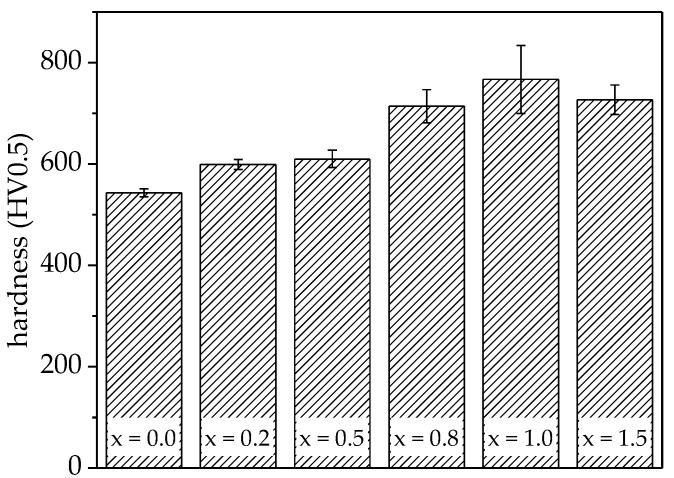
Microhardness of arc-melted AlCoCrFeNiTi_x_ samples. Microhardness increases with titanium content, reaching a maximum of 770 HV0.5 for the equimolar alloy.

The influence of the titanium content on the average microhardness HV0.5 was investigated. The results are summarised in Figure 4.

For the alloy without titanium (x = 0.0), an average microhardness of 550 HV0.5 was measured. With increasing titanium content, an increase of microhardness was observed, reaching a maximum for the equimolar composition with a microhardness of 770 HV0.5. The increasing hardness indicated the formation of additional phases, which was in accordance with microstructural investigations and phase analyses. Furthermore, solid solution strengthening contributes to an increase of hardness—an increase of lattice parameters could be proven for the bcc (B2) and fcc (A1) phases. However, for the alloy with the highest titanium content (x = 1.5), a reduced microhardness of 730 HV0.5 was measured, which might be a result of the increased presence of cavities and shrinkage porosity in that alloy.

**Figure 5:**
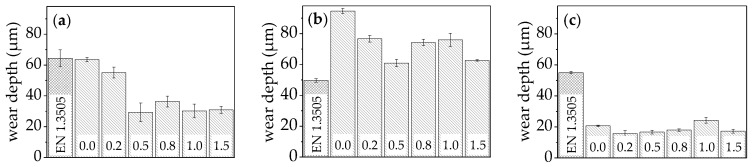
Wear depths of bearing steel EN 1.3505 and AlCoCrFeNiTi_x_ in: (**a**) Ball-on-disk; (**b**) Oscillating wear and (**c**) Scratch tests. The wear depths of AlCoCrFeNiTi_x_ are decreased in comparison to EN 1.3505, except under oscillating wear conditions. Overall, the best results are obtained for x = 0.5.

The wear behaviour was investigated under adhesive, oscillating, and abrasive wear conditions in ball-on-disk, oscillating wear, and scratch tests. The results are summarised in Figure 5.

The investigation of the wear behaviour in the ball-on-disk test revealed a high wear depth for the alloy without titanium (x = 0.0). With the addition of titanium (x = 0.2), a slight decrease of wear depth could be achieved. Further increase to x = 0.5 resulted in a distinct decrease of wear depth. The alloy x = 0.5 exhibited a multiphase character, only comprising cubic phases. In comparison to the alloys with lower titanium content, the microhardness was increased, which enhanced wear resistance in ball-on-disk tests. However, further increase of titanium content did not cause a reduction of wear depth or an improvement of the wear resistance. Phase analyses revealed the formation of additional complex phases reducing wear resistance. All samples containing titanium exhibited a lower wear depth in comparison with the bearing steel EN 1.3505, and hence a higher wear resistance in the ball-on-disk test.

In the oscillating wear tests, the highest wear depth was measured for the alloy without titanium (x = 0.0). Adding minor amounts of titanium resulted in a decrease of the wear depth. For the sample x = 0.5, the lowest wear depth was measured, showing that a multiphase character only comprising cubic phases was advantageous under oscillating wear condition. The further addition of titanium led to an increase of the wear depth. Additional tetragonal or cc phases did not contribute to an improvement of wear resistance. For the alloy with the highest titanium content (x = 1.5), a low wear depth was measured, which was in the range of the alloy with x = 0.5. In comparison with bearing steel EN 1.3505, all investigated samples exhibited a higher wear depth, and thereby lower wear resistance under oscillating wear conditions.

**Figure 6:**
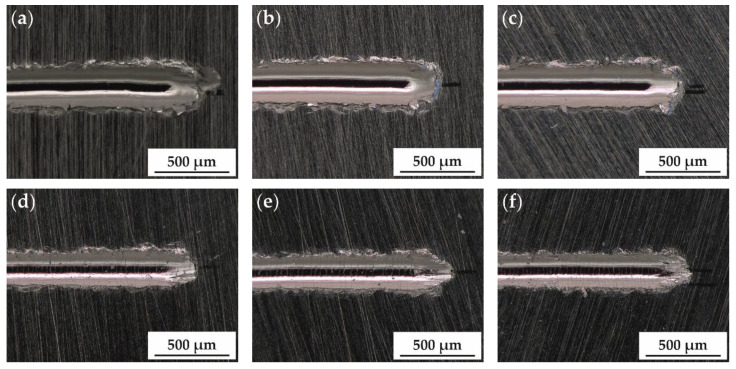
Surface of the arc-melted AlCoCrFeNiTix samples: (**a**) x = 0.0; (**b**) x = 0.2; (**c**) x = 0.5; (**d**) x = 0.8; (**e**) x = 1.0; (**f**) x = 1.5 after progressive mode scratch test. Ductile behaviour occurs for x ≤ 0.5, whereas cracks or spalling of material are visible for x ≥ 0.8.

Under abrasive tribological conditions in the scratch tests, the lowest wear depth was measured for the sample with a titanium content of x = 0.2. With an increase in titanium content, the wear depth slightly increased. The highest wear depth was measured for the equimolar alloy (x = 1.0). In comparison to the bearing steel EN 1.3505, all investigated samples exhibit a distinctly lower wear depth and thereby higher wear resistance. The wear tracks of all samples were investigated by optical microscopy. In Figure 6 images of the sample surface where the highest load was applied in the progressive mode scratch test are shown.

The investigation of the surface after scratch test under abrasive conditions reveals no cracks or spalling of material along the main scratch for the sample without titanium (x = 0.0) and the two samples with the lowest titanium content (x = 0.2; x = 0.5). However, spalling of material and distinct secondary cracks perpendicular to the main scratch can be observed for all samples with a titanium content exceeding x = 0.5. The formation of secondary cracks and spalling of material indicated the brittle behaviour of these alloys, which is caused by the formation of additional, complex phases—tetragonal, cc and a hexagonal Laves phase for a titanium content x ≥ 0.8. These phases possess low numbers of slip systems, which results in reduced ductility.

## 4. Summary and Conclusions

The influence of the alloying element titanium was studied in detail in the alloy system AlCoCrFeNiTi_x_. For the alloy without titanium (x = 0.0), a single chemically ordered bcc phase with B2 structure is formed. In contrast, a multiphase microstructure was revealed for all titanium-containing alloys. With an increase of titanium content, an increase of heterogeneity of the microstructure is observed. Furthermore, the hardness can be distinctly increased, whereas the maximum hardness is achieved for the equimolar composition. Phase analyses prove the formation of two major bcc phases for all titanium-containing samples. One chemically ordered bcc phase with B2 structure and one chemically disordered bcc phase with A2 structure is formed. With an increase in titanium content, additional phases occur. For x ≥ 0.5 a minor fraction of an fcc phase was detected. A further increase in titanium content results in additional, more complex phases. This could also be proved by analysing the solidification behaviour. Analyses of the lattice parameters revealed a shift to bigger values with increasing titanium content, especially for the bcc (B2) and the fcc (A1) phase. The alloy system AlCoCrFeNiTi_x_ exhibits an increased wear resistance in comparison with the bearing steel EN 1.3505, except under oscillating wear conditions.

Correlations between phase composition, microstructure, and wear resistance can be concluded. Microstructure design for high wear resistance requires cubic phases. Hereby, a multiple bcc/fcc phase character exhibits an advantageous behaviour. Complex phases (cc and tetragonal) increase the hardness, but should be avoided in order to achieve a high wear resistance, as the presence of these phases causes increased brittleness. AlCoCrFeNiTi_0.5_ is a promising candidate for wear protection applications in both bulk and coating materials.

## Figures and Tables

**Table 1:** Wear test parameters.

Ball-on-Disk Test		Oscillating Wear Test		Scratch Test	
Force	20 N	Force	26 N	Mode	progressive
Radius	5 mm	Frequency	40 Hz	Force	1–200 N
Speed	96 RPM	Time	900 s	Speed	2.5 mm/min
Cycles	15916	Amplitude	0.5 mm	Length	5 mm
Counter body	Al_2_O_3_	Counter body	Al_2_O_3_	Tip	Rockwell C
Diameter	6 mm	Diameter	10 mm	Radius	200 µm

**Table 2:** Mean chemical composition (in at.%) of AlCoCrFeNiTi_x_ samples, measured by EDS and nominal values. The measured chemical compositions are in good agreement with the target compositions.

x		Al	Co	Cr	Fe	Ni	Ti
**0.0**	nominal	20.0	20.0	20.0	20.0	20.0	20.0
	measured	21.3	19.4	19.9	19.9	19.5	0.0
**0.2**	nominal	19.2	19.2	19.2	19.2	19.2	3.9
	measured	20.3	18.9	19.0	19.2	18.7	3.9
**0.5**	nominal	18.2	18.2	18.2	18.2	18.2	9.1
	measured	20.0	17.8	17.4	17.6	18.2	9.0
**0.8**	nominal	17.2	17.2	17.2	17.2	17.2	13.8
	measured	19.0	16.7	16.8	16.7	17.3	13.5
**1.0**	nominal	16.7	16.7	16.7	16.7	16.7	16.7
	measured	17.9	16.2	16.7	16.6	16.4	16.3
**1.5**	nominal	15.4	15.4	15.4	15.4	15.4	23.1
	measured	16.7	15.2	15.4	15.3	15.6	22.0

**Table 3:** Summary of phases detected by XRD analyses for arc-melted AlCoCrFeNiTi_x_ samples.

x	Phase	Struktur-Bericht	Lattice	Structure	Pearson Symbol	Space Group	Lattice Parameter (Å)
**0.0**	PP	B2	bcc	CsCl	cP2	Pm$\bar{3}$m (221)	2.88
**0.2**	PP	B2	bcc	CsCl	cP2	Pm$\bar{3}$m (221)	2.89
	IP	A2	bcc	W	cI2	Im$\bar{3}$m (229)	2.87
**0.5**	PP	B2	bcc	CsCl	cP2	Pm$\bar{3}$m (221)	2.92
	IP	A2	bcc	W	cI2	Im$\bar{3}$m (229)	2.87
	CW	A1	fcc	Cu	cF4	Fm$\bar{3}$m (225)	5.82
**0.8**	PP	B2	bcc	CsCl	cP2	Pm$\bar{3}$m (221)	2.92
	IP	A2	bcc	W	cI2	Im$\bar{3}$m (229)	2.87
	CW	A1	fcc	Cu	cF4	Fm$\bar{3}$m (225)	5.85
	CW	C4	tetragonal	TiO_2_	tP6	P4_2_/mnm (136)	a = 8.80 c = 4.56
**1.0**	PP	B2	bcc	CsCl	cP2	Pm$\bar{3}$m (221)	2.93
	IP	A2	bcc	W	cI2	Im$\bar{3}$m (229)	2.88
	CW	A1	fcc	Cu	cF4	Fm$\bar{3}$m (225)	5.87
	CW	A12	cc	α-Mn	cI58	I$\bar{4}$3m (217)	8.92
**1.5**	PP	B2	bcc	CsCl	cP2	Pm$\bar{3}$m (221)	2.94
	IP	A2	bcc	W	cI2	Im$\bar{3}$m (229)	2.88
	CW	A1	fcc	Cu	cF4	Fm$\bar{3}$m (225)	5.87
	CW	C14	hexagonal	MgZn_2_	hP12	P6_3_/mmc (194)	a = 4.80 c = 7.81
